# Course and characteristics of work disability 3 years before and after lumbar spine decompression surgery– a national population-based study

**DOI:** 10.1038/s41598-018-30211-4

**Published:** 2018-08-07

**Authors:** Thomas E. Dorner, Magnus Helgesson, Kerstin Nilsson, Konstantinos A. Pazarlis, Annina Ropponen, Pia Svedberg, Ellenor Mittendorfer-Rutz

**Affiliations:** 1 Medical University of Vienna, Centre for Public Health, Department for Social and Preventive Medicine, Kinderspitalgasse 15/I, 1090 Wien, Austria; 2 Karolinska Institutet, Department of Clinical Neuroscience, Division of Insurance Medicine, Berzeliusv. 3, 171 77, Stockholm, Sweden; 3 Department of Surgical Sciences, Division of Orthopedics, Uppsala University Hospital, Akademiska sjukhuset ing 70, SE-751 85, Uppsala, Sweden; 4 Finnish Institute of Occupational Health, P.O. Box 18, 00390 Helsinki, Finland

## Abstract

Despite decompression surgery being a widespread intervention for patients with dorsopathies (i.e. back pain) affecting the lumbar spine, the scientific knowledge on patterns and characteristics of work disability before and after the surgery is limited. Sickness absence (SA) and disability pension (DP) were examined three years before and after surgery in 8558 patients aged 25–60 years who underwent lumbar spine decompression surgery in Sweden. They were compared to individuals with diagnosed dorsopathies but no surgery and individuals from the general population as matched comparison groups. According to Group Based Trajectory models, in patients with decompression surgery, 39% had low levels of SA/DP during the entire study period and 15% started with low levels of SA/DP, which increased in the year before, and declined to almost zero in the second year after surgery. Three trajectory groups (12%, 17%, and 18%) started at different levels of SA/DP, which increased in the years before, and declined in the third year after surgery. The trajectory groups in the comparison groups showed lower levels of work disability. Sex, education, and the use of antidepressants and analgesics the year before surgery played an important role to explain the variance of trajectory groups in patients with surgery.

## Introduction

Worldwide, back problems, and in specific dorsopathies (i.e. back pain) constitute one of the most critical public health problems^1,2^. The disorder ranks among the most frequent causes of functional impairment including short and permanent work disability (i.e. sickness absence, SA, and disability pension, DP) and covers a clinically heterogeneous patient group, ranging from unspecific pain to more specific disorders^1–4^. Treatment and rehabilitation measures for individuals with more severe dorsopathy seeking help in the healthcare system range from the patient’s educational level, exercise therapy, pharmacological treatment, physio- and psychotherapy to spinal manipulation and decompression surgery in some cases^1–6^. Surgical decompression in the lumbar spine, which can be combined with fusion surgery, is recommended for the treatment of symptomatic spinal stenosis combined with degenerative lumbar spondylolisthesis, when other medical and interventional treatments did not lead to success^7^. In Sweden, approximately 2% of individuals undergo a lumbar spine surgery in their life time^4^. There is an ongoing debate when patients with certain degenerative or intervertebral disc disorders should undergo surgery or conservative therapy^6,8^. Previous studies with clinical characteristics as endpoints have shown inconclusive results or trends of better clinical results in patients with surgery, especially in the short-term follow-up period^9,10^.

The prognosis after decompression surgery in terms of regaining functional capacity including work capacity is strongly influenced by socio-demographic and clinical factors. Socio-economic differences e.g. have been reported to affect work disability in general^11^, and this is especially pronounced regarding pain-related SA. Clinical factors elevating the risk for work disability in patients with dorsopathy include the symptom severity and comorbid disorders particularly common mental disorders (CMDs, i.e. depressive and anxiety disorders), which are strongly interlinked with dorsopathy^3,12,13^. In Europe, up to one third of subjects with chronic pain (the majority with back pain) have a comorbid depression or anxiety^1^.

Despite the overwhelming evidence related to the effect of different rehabilitation and treatment methods on the vocational prognosis of individuals with dorsopathies, scientific knowledge on patterns of work disability before and after spine surgery in general and decompression surgery in specific is limited or conflicting^4,14–17^. Existing research in this respect is mainly based on small clinical samples often with retrospective, self-reported data and thereby inherently challenged by methodological shortcomings and has seldom considered socio-demographic and a range of clinical factors in related associations^15^.

Moreover, studies on the effect of the most frequently occurring comorbid condition, i.e. a preoperative CMD, on work disability after spine surgery are particularly sparse^14^. Studies comparing patterns (trajectories) in patients with dorsopathies and decompression surgery with a group of patients without surgery are entirely missing to the best of our knowledge. In order to provide clinically relevant information, studies in this field have to be sufficiently powered and use tailor-made methodology to capture the prognostic heterogeneity after decompression surgery^18–21^.

By using national registers covering all patients with decompression surgery in Sweden, the aims of this study were to 1) identify trajectories of work disabilities in terms of SA/DP in patients with dorsopathies prior and after decompression surgery; 2) compare these findings to patients with dorsopathies without surgery and to individuals without dorsopathies. Furthermore, we aimed to elucidate associations of socio-demographic and clinical factors in the identified trajectory groups of work disability in patients with decompression surgery.

## Results

### Study Populations

Descriptive statistics of the socio-demographic matching factors of cases and individuals in both comparison groups are presented in Table 1. Somewhat more than half of the cases were men and the majority was between 35 and 54 years of age with a medium educational level (i.e. high school) and born in Sweden. The majority of cases had radiculopathies (62.7%), followed by spondylopathies (33.8%) and other dorsopathies (3.5%). In total, 18.5% had an additional fusion surgery during the inclusion period (2008–2010).

**Table 1:** Descriptive data of matched variables among 8558 individuals with dorsopathy diagnosed in specialized health care as well as decompression surgery 2008–2010 in Sweden.

Sociodemographic factors	N (%)
***Sex***	
Men	4561 (53.3)
Women	3997 (46.7)
***Age***	
25–34	1328 (15.5)
35–44	2601 (30.4)
45–54	2572 (30.1)
55–60	2057 (24.0)
***Educational level*** (***years***)	
Compulsory school (<9)	1503 (17.6)
High school (10–12)	4594 (53.7)
College or university (>12)	2461 (28.8)
***Region of birth country***	
Sweden	7096 (82.9)
Nordic countries (except Sweden)	370 (4.3)
Europe (Except Nordic countries)	202 (2.4)
Outside Europe	890 (10.4)

Other socio-demographic and clinical factors for cases and subjects in the comparison groups are presented in Table 2. The majority of both cases and individuals in the comparison groups lived in big or medium sized cities, was married or cohabitating with children living at home and had not been unemployed in the year before inclusion. Considerable differences in the three groups emerged, however, with regard to the clinical factors: almost all cases were work disabled three years before or three years after inclusion, which was not the case for individuals in comparison groups 1 and 2. About two thirds of cases and of the individuals in the comparison group 1 and half of the individuals in the comparison group 2 had specialized health care due to other somatic disorders. During the year before inclusion the proportion of individuals with prescribed analgesics was clearly higher for the cases than for the subjects in the comparison groups. The majority of cases had low doses of analgesics, though. The occurrence of mental disorders and prescription of anxiolytics, sedatives and antidepressants was similar in cases and patients in the comparison group 1, but differed to individuals in the comparison group 2.

**Table 2:** Descriptive data of 8558 individuals with decompression surgery 2008–2010 and two matched comparison groups: (a) individuals with dorsopathies, but no surgery 2008–2010 and (b) the general population without dorsopathies or surgery 2001–2013.

	Surgery (N = 8558)	Comparison group 1: Dorsopathy, no surgery (N = 8558)	Comparison group 2: General population (N = 8558)
**Sociodemographic factors**			
***Type of living area***			
Big cities	3314 (38.7)	3405 (39.8)	3122 (36.5)
Medium sized cities	2918 (34.1)	2852 (33.3)	2966 (34.7)
Small town	2326 (27.2)	2301 (26.9)	2470 (28.9)
***Family situation***			
Married or cohabiting/with no children at home	1461 (17.1)	1372 (16.0)	1351 (15.8)
Married or cohabiting/with children at home	3744 (43.7)	3626 (42.4)	3642 (42.6)
Single with no children at home	2514 (29.4)	2740 (32.0)	2858 (33.4)
Single with children at home	839 (9.8)	820 (9.6)	707 (8.3)
***Unemployment in the year before inclusion***			
No unemployment	7810 (91.3)	7692 (89.9)	7793 (91.1)
1–179 days	555 (6.5)	635 (7.4)	555 (6.5)
≥180 days	193 (2.3)	231 (2.7)	210 (2.5)
**Clinical factors**			
***Work disability*** ^*a*^ *3 years before inclusion*	6046 (70.7)	4709 (55.0)	2333 (27.3)
**Work disability 3 years after inclusion**	7605 (88.9)	5041 (58.9)	2282 (26.7)
***Previous and current dorsopathies***			
Dorsopathies^b^ 3 years before inclusion	7454 (87.1)	989 (11.6)	—
**Fusion surgery at inclusion**	1580 (18.5)	—	—
*Other Somatic diagnoses* ^***c***^ *3 years before and at inclusion*	5720 (66.8)	5959 (69.6)	4215 (49.3)
***Analgesics*** ^*d*^ ***1 years before inclusion***			
No Analgesics	1644 (19.2)	4501 (52.6)	7615 (89.0)
Low dose (0–0.5 DDD^e^)	4910 (57.4)	3130 (36.6)	806 (9.4)
Medium dose (0.5–1.5 DDD)	1575 (18.4)	635 (7.4)	101 (1.2)
High dose (>1.5 DDD)	429 (5.0)	292 (3.4)	36 (0.4)
***Previous mental disorders***			
*Common mental disorders* ^***f***^ *3 years before and at inclusion*	526 (6.2)	677 (7.9)	291 (3.4)
*Other mental diagnoses* ^***g***^ *3 years before and at inclusion*	446 (5.2)	581 (6.8)	331 (3.9)
***Anxiolytics*** ^***h***^ ***1 year before inclusion***			
*No Anxiolytics*	7341 (85.8)	7546 (88.2)	8161 (95.4)
*Low dose* (*0–0*.*5 DDD*)	1048 (12.3)	820 (9.6)	323 (3.8)
*Medium dose* (*0*.*5–1*.*5 DDD*)	113 (1.3)	116 (1.4)	50 (0.6)
*High dose* (>*1*.*5 DDD*)	56 (0.7)	76 (0.9)	24 (0.3)
***Hypnotics/Sedatives*** ^***i***^ ***1 year before inclusion***			
*No Hypnotics/Sedatives*	7161 (83.7)	7277 (85.0)	7981 (93.3)
*Low dose* (*0–0*.*5 DDD*)	787 (9.2)	744 (8.7)	341 (4.0)
*Medium dose* (*0*.*5–1*.*5 DDD*)	396 (4.6)	360 (4.2)	162 (1.9)
*High dose* (>*1*.*5 DDD*)	214 (2.5)	177 (2.1)	74 (0.9)
***Antidepressants*** ^***j***^ ***1 year before inclusion***			
*No Antidepressants*	6846 (80.0)	7028 (82.1)	7801 (91.2)
*Low dose* (*0–0*.*5 DDD*)	727 (8.5)	606 (7.1)	251 (2.9)
*Medium dose* (*0*.*5–1*.*5 DDD*)	633 (7.4)	601 (7.0)	350 (4.1)
*High dose* (>*1*.*5 DDD*)	352 (4.1)	323 (3.8)	156.8)

^a^Work disability was defined as having had at least one day of reimbursed sickness absence or disability pension in this period.

^b^Spondylopathies, (International Classification of Diseases version 10 (ICD-10): M47.8, M47.9, M47.9 K, M48.0, M48.0 K, M48.8, M48.8 K and M48.8 W), Radiculopathies (ICD-10: M51.1, M51.1 K and M54.1) and other dorsopathies (ICD-10: M54.3, M54.4, M54.5, M53.8 and M53.9).

^c^All diagnoses except mental diagnoses (F00-F99), dorsopathic diagnoses (M40-M54), uncomplicated delivery (ICD-10: O80), external causes of morbidity and mortality (ICD-10: V01-Y98), factors influencing health status and contact with health services (ICD-10: Z00-Z99), and codes for special purposes (ICD-10: U00-U89).

^d^Anatomical therapeutic chemical classification system (ATC) codes: N02A, N02B.

^e^Defined daily dose (DDD) is the assumed average maintenance dose per day for a drug used for its main indication in adults.

^f^ICD-10: F32, F33 and F40- F43.

^g^ICD-10: F00-F31, F34-F39 and F44-F99.

^h^ATC Code N05B.

^i^ATC Code N05C.

^j^ATC code: N06.

### Patterns of work disability

**Figure 1:**
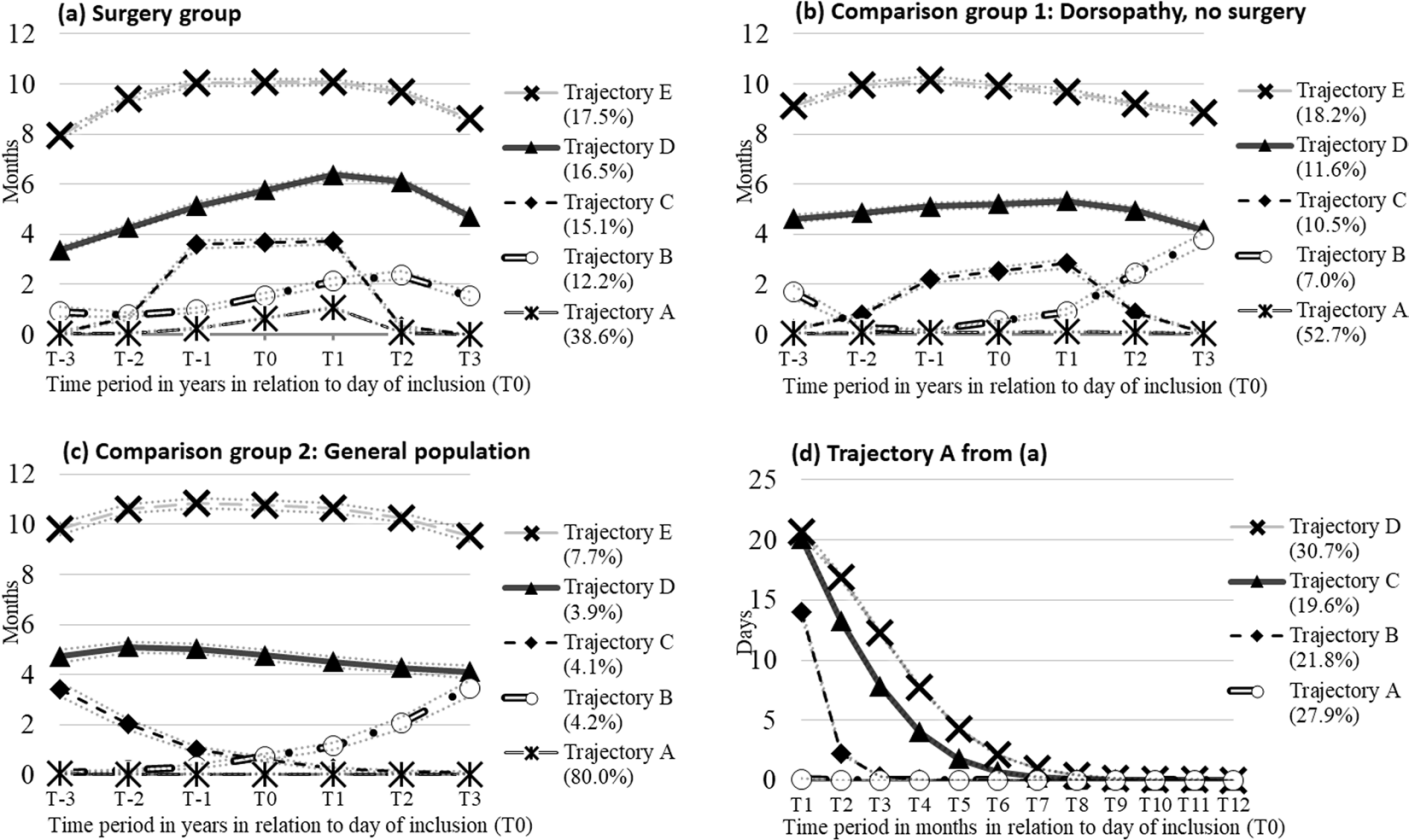
(**a**–**d**) Trajectories of work disability among 8558 individuals with decompression surgery 2008–2010 **a**, matched comparison group 1 comprising individuals with dorsopathies, but no surgery 2008–2010 **b**, matched comparison group 2 including individuals in the general population without diagnosed dorsopathies 2001–2013 **c** and 3306 individuals following trajectory A in (**a**) during the first year after surgery (**d**)^a^ a The value for T0 was calculated as the mean value for T-1 and T1 and inserted here for pedagogic purposes.

In patients with surgery five groups of different trajectories could be identified (Fig. 1a). The largest proportion (39%) of individuals comprised trajectory group A. They had almost no SA/DP before surgery, only in the year after surgery they had a mean of one month of SA/DP, which decreased to almost zero after the first year. Trajectory group B (12%) was characterized by low levels of SA/DP before surgery, an increase up to two years after surgery, after which they levelled off. Trajectory group C (15% of the cases) started with very low levels of SA/DP until one year before surgery, when levels increased to four months of SA/DP, declining to almost no SA/DP levels after the first year following surgery. Trajectory group D showed medium SA/DP levels three years before surgery and steadily increased to six SA/DP months in the year after surgery, and a somewhat lower level in the third year after surgery. Finally, 17% of patients (trajectory group E) started with a high level of SA/DP (eight months), increased to ten months in the year before surgery, stayed at this high level until the year after surgery, and decreased to eight months in the third year after surgery.

Further scrutiny of trajectory group A during the first year after surgery revealed four sub-groups (Fig. 1d). One group (28%) had very low or no SA/DP days per month, while three groups had decreasing SA/DP days after the surgery. The patterns began at different levels (15 and 20 days per month) and reached zero SA/DP days after 3, 6 and 8 months respectively (trajectory B, C, and D).

Patterns and proportions of work disability in patients in the comparison groups (Fig. 1b,c) were comparable to those of the cases (Fig. 1a), with the following main exception: the proportion of the group with the lowest levels of work disability (trajectory A) was higher in the groups without surgery (53% and 80%, respectively vs 39%) and there was no increase in SA/DP months around T0;

### Factors associated with trajectories in patients with decompression surgery

Table 3 shows the distribution of socio-demographic and clinical characteristics across the different trajectory groups in the cases. All analyzed variables were significantly associated with the trajectory groups except for unemployment in the year before surgery. The full model, with all socio-demographic and clinical factors explained 34% of the variance between the trajectory groups. According to the differences in R^2^, sex, education, antidepressants and analgesics the year preceding the surgery had a more important role than other factors in the full model.

**Table 3:** Characteristics related to different trajectories of work disability among 8558 individuals with a diagnosis of dorsopathy in specialized health care and a decompression surgery 2008–2010 in Sweden.

	Trajectory group A	Trajectory group B	Trajectory group C	Trajectory group D	Trajectory group E	Chi^2^ (p)	R^2^difference
	N (%)	N (%)	N (%)	N (%)	N (%)		(R^2^ = 0.342)
**All**	3306 (38.6)	1043 (12.2)	1295 (15.1)	1414 (16.5)	1500 (17.5)		
**Sociodemographic factors**							
***Sex***							
*Men*	2106 (46.2)	529 (11.6)	698 (15.3)	632 (13.9)	596 (13.1)	192.6 (<0.001)	0.016
*Women*	1200 (30.0)	514 (12.9)	597 (14.9)	782 (19.6)	904 (22.6)		
***Age***							
*25–34*	656 (49.4)	170 (12.8)	244 (18.4)	166 (12.5)	92 (6.9)	89.5 (<0.001)	<0.01
*35–44*	1190 (45.8)	345 (13.3)	417 (16.0)	366 (14.1)	283 (10.9)		
*45–54*	893 (34.7)	302 (11.7)	394 (15.3)	462 (18.0)	521 (20.3)		
*55–60*	567 (27.6)	226 (11.0)	240 (11.7)	420 (20.4)	604 (29.4)		
***Educational level*** (***years***)							
*Compulsory school* (<*9*)	381 (25.4)	144 (9.6)	222 (14.8)	305 (20.3)	451 (30.0)	423.6 (<0.001)	0.037
*High school* (*10–12*)	1591 (34.6)	595 (13.0)	773 (16.8)	801 (17.4)	834 (18.2)		
*College or university* (>*12*)	1334 (54.2)	304 (12.4)	300 (12.2)	308 (12.5)	215 (8.7)		
***Type of living area***							
*Big cities*	1467 (44.3)	409 (12.3)	474 (14.3)	459 (13.9)	505 (15.2)	72.1 (<0.001)	<0.01
*Medium sized cities*	1085 (37.2)	338 (11.6)	455 (15.6)	512 (17.6)	528 (18.1)		
*Small town*	754 (32.4)	296 (12.7)	366 (15.7)	443 (19.1)	467 (20.1)		
***Family situation***							
*Married or cohabiting*/*with no children at home*	435 (29.8)	167 (11.4)	202 (13.8)	296 (20.3)	361 (24.7)	31.4 (<0.001)	<0.01
*Married or cohabiting*/*with children at home*	1712 (45.7)	495 (13.2)	581 (15.5)	533 (14.2)	423 (11.3)		
*Single or cohabitating*/*with no children at home*	894 (35.6)	268 (10.7)	393 (15.6)	434 (17.3)	525 (20.9)		
*Single/cohabitation with children*	265 (31.6)	113 (13.5)	119 (14.2)	151 (18.0)	191 (22.8)		
***Region of birth***							
*Sweden*	2794 (39.4)	913 (12.9)	1103 (15.5)	1160 (16.4)	1126 (15.9)	109.6 (<0.001)	<0.01
*Nordic countries* (*except Sweden*)	116 (31.4)	39 (10.5)	43 (11.6)	74 (20.0)	98 (26.5)		
*Europe* (*except Nordic countries*)	76 (37.6)	23 (11.4)	36 (17.8)	27 (13.4)	40 (19.8)		
*Outside Europe*	320 (36.0)	68 (7.6)	113 (12.7)	153 (17.2)	236 (26.5)		
**Work-related factors**							
***Unemployment in the year*** ***before inclusion***							
*No unemployment*	3060 (39.2)	946 (12.1)	1179 (15.1)	1244 (15.9)	1381 (17.7)	1.7 (0.43)	<0.01
*1–179 days*	175 (31.5)	75 (13.5)	95 (17.1)	119 (21.4)	91 (16.4)		
≥*180 days*	71 (36.8)	22 (11.4)	21 (10.9)	51 (26.4)	28 (14.5)		
**Previous and current dorsopathies**							
*Dorsopathies* ^*a*^ *3 years before inclusion*	2784 (37.4)	883 (11.9)	1179 (15.8)	1271 (17.1)	1337 (17.9)	5.0 (0.03)	<0.01
***Dorsopathic diagnoses at inclusion***							
Spondylopathies	790 (27.3)	321 (11.1)	341 (11.8)	612 (21.2)	828 (28.6)	71.4 (<0.001)	<0.01
Radiculopathies	2462 (45.9)	691 (12.9)	905 (16.9)	727 (13.6)	576 (10.7)		
Other dorsopathies	53 (17.6)	31 (10.3)	49 (16.2)	74 (24.5)	95 (31.5)		
*Fusion surgery at inclusion*	300 (19.0)	132 (8.4)	255 (16.1)	391 (24.8)	502 (31.8)	90.9 (<0.001)	<0.01
*Other somatic diagnoses* ^*b*^ *3 years before and at inclusion*	1840 (32.2)	769 (13.4)	837 (14.6)	1052 (18.4)	1222 (21.4)	79.3 (<0.001)	<0.01
***Analgesics*** ^***c***^ ***1 year before inclusion***							
*No DDD* ^*d*^	862 (52.4)	192 (11.7)	184 (11.2)	208 (12.7)	198 (12.0)	273.7 (<0.001)	0.023
*Low dose* (*0–0*.*5 DDD*)	2099 (42.8)	648 (13.2)	788 (16.1)	731 (14.9)	644 (13.1)		
*Medium dose* (*0*.*5–1*.*5 DDD*)	307 (19.5)	171 (10.9)	283 (18.0)	381 (24.2)	433 (27.5)		
*High dose* (*>1*.*5 DDD*)	38 (8.9)	32 (7.5)	40 (9.3)	94 (21.9)	225 (52.5)		
**Previous mental disorders**							
*Common mental disorders* ^***e***^ *3 years before and at inclusion*	69 (13.1)	41 (7.8)	47 (8.9)	109 (20.7)	260 (49.4)	31.6 (<0.001)	<0.01
*Other mental diagnoses* ^***f***^ *3 years before and at inclusion*	54 (12.1)	40 (9.0)	32 (7.2)	91 (20.4)	229 (51.4)	58.9 (<0.001)	<0.01
***Anxiolytics/sedatives*** ^***g***^ ***1 year before inclusion***							
*No DDD*	3015 (41.1)	901 (12.3)	1147 (15.6)	1195 (16.3)	1083 (14.8)	15.6 (0.001)	<0.01
*Low dose* (*0–0*.*5 DDD*)	280 (26.7)	129 (12.3)	145 (13.8)	194 (18.5)	300 (28.6)		
*Medium dose* (*0*.*5–1*.*5 DDD*)	8 (7.1)	7 (6.2)	2 (1.8)	21 (18.6)	75 (66.4)		
*High dose* (*>1*.*5 DDD*)	3 (5.4)	6 (10.7)	1 (1.8)	4 (7.1)	42 (75.0)		
***Hypnotics*** ^***h***^ ***1 year before inclusion***							
No DDD	3055 (42.7)	900 (12.6)	1154 (16.1)	1089 (15.2)	963 (13.5)	70.8 (<0.001)	<0.01
*Low dose* (*0–0*.*5 DDD*)	194 (24.7)	90 (11.4)	108 (13.7)	178 (22.6)	217 (27.6)		
*Medium dose* (*0*.*5–1*.*5 DDD*)	41 (10.4)	45 (11.4)	25 (6.3)	99 (25.0)	186 (47.0)		
*High dose* (>*1*.*5 DDD*)	16 (7.5)	8 (3.7)	8 (3.7)	48 (22.4)	134 (62.6)		
***Antidepressants*** ^***i***^ ***1 year before inclusion***							
*No DDD*	3001 (43.8)	842 (12.3)	1097 (16.0)	1026 (15.0)	880 (12.9)	135.9 (<0.001)	0.011
*Low dose* (*0–0*.*5 DDD*)	175 (24.1)	89 (12.2)	110 (15.1)	152 (20.9)	201 (27.7)		
*Medium dose* (*0*.*5–1*.*5 DDD*)	91 (14.4)	78 (12.3)	69 (10.9)	154 (24.3)	241 (38.1)		
*High dose* (>*1*.*5 DDD*)	39 (11.1)	34 (9.7)	19 (5.4)	82 (23.3)	178 (50.6)		

^a^Spondylopathies, (International Classification of Diseases version 10 (ICD-10): M47.8, M47.9, M47.9 K, M48.0, M48.0 K, M48.8, M48.8 K and M48.8 W), Radiculopathies (ICD-10: M51.1, M51.1 K and M54.1) and other Dorsopathies (ICD-10: M54.3, M54.4, M54.5, M53.8 and M53.9).

^b^All diagnoses except mental diagnoses (F00-F99), dorsopathic diagnoses (M40-M54), uncomplicated delivery (ICD-10: O80), external causes of morbidity and mortality (ICD-10: V01-Y98), factors influencing health status and contact with health services (ICD-10: Z00-Z99), and codes for special purposes (ICD-10: U00-U89).

^c^Anatomical therapeutic chemical classification system (ATC) codes: N02A, N02B.

^d^Defined daily dose (DDD) is the assumed average maintenance dose per day for a drug used for its main indication in adults.

^e^ICD-10: F32, F33 and F40- F43.

^f^ICD-10: F00-F31, F34-F39 and F44-F99.

^g^ATC Code N05B.

^h^ATC Code N05C.

^i^ATC code: N06.

Individuals with a low amount of work disability (trajectory A) were more likely to be men, younger, with a higher educational level (i.e. college or university), living in big cities, married or cohabitating with children living at home, and to have no prescription of analgesics, anxiolytics, hypnotics or antidepressants one year before surgery. The trajectory group B comprised individuals with no distinct pattern of characteristics. Patients with work disability mainly in the year before and the year after surgery (trajectory C), tended to be younger with a migration background from Europe (except Nordic countries), and a low or medium dose of analgesics one year before surgery. Patients with high amount of work disability (trajectories D and E) were more frequently female, older, with compulsory educational level only, living in small towns, married or cohabitating with no children living at home or single. Moreover, they were likely to have migration background from Nordic countries or from outside Europe. Regarding clinical characteristics, patients in trajectory groups D and E had often fusion surgery at inclusion, health care due to somatic diagnoses (other than dorsopathies) three years before and at inclusion, medium or high doses of analgesics one year before inclusion, specialized health care due to mental disorders three years before and at inclusion, and low to high doses of anxiolytics, hypnotics, and antidepressants one year before inclusion. Especially the factors associated with mental diagnoses were much more pronounced in individuals of trajectory groups D and E as compared to the other trajectory groups.

## Discussion

Patterns of work disability were found to be very heterogeneous in patients with dorsopathy and a decompression surgery. We found five different groups of trajectories regarding SA and DP. All of them had a peak of SA/DP around the time of surgery. Still, the groups of trajectories started on different levels of SA/DP three years before surgery (ranging from zero to eight months per year). Within these groups, SA/DP started to increase at different times (ranging from three years before surgery to the year of surgery), and started to decrease at different times (ranging from the year of surgery to the second year after surgery). Sex, education, and the use of antidepressants and analgesics the year before surgery determined the heterogeneity of work disability in patients with surgery.

The largest proportion of cases with decompression surgery (trajectory group A) had very low or no levels of work disability during the entire observation period with a peak during the year following the surgery. During this year, most of the patients had either no work disability or decreasing SA/DP levels already after three months following surgery. Patients in this trajectory group were predominantly younger, male with college or university education and no drug treatment with analgesics, anxiolytics, hypnotic or antidepressants the year before surgery. In general, young educated men represent the group of patients with the best prognosis in terms of work disability^11^. Moreover, high education^22^, lower severity of symptoms and lower work disability before surgery^23^ have been shown to predict a positive outcome in terms of work disability after spine surgery in previous studies, with some conflicting results in terms of age and sex^22,23^. Interestingly, in this trajectory group, many patients did not have prescribed and dispensed analgesic drugs the year prior to the surgery. However, according to recommendations, decompression surgery is performed as the primary option of therapy in rare acute cases of severe neurological complications like cauda equine syndrome^5,6^, and more often electively, in patients whose symptoms do not sufficiently respond to other medical and interventional treatment options^6,7^. Since many of the patients in this trajectory group did not receive prescribed analgesic medication the year before surgery, they might have only consumed over-the-counter analgesics and/or decompression surgery might have been the first choice of elective therapy in many of those cases.

Trajectory group C (15% of patients with decompression surgery) comprised probably the group of patients with the highest gain in work ability after decompression surgery. They had a high level of work disability two years before surgery and could decrease work disability to almost zero within the first year after surgery. This group of patients included predominantly young people of both sexes with no high dosage of analgesics prior to surgery. Thus, they were a group of patients where a better prognosis could be expected, anyway. Here, it is important to mention that the same patterns of trajectory group C were even found in comparison group 1. Future studies with a more pronounced experimental design are warranted to elucidate the long-term gain in work ability following decompression surgery.

We found three trajectory groups in the surgery cases (B, D, and E) with similar patterns of work disability, i.e. increasing SA/DP at different levels up to the surgery. Work disability in these groups increased most likely due to the steady progression of the spine disorders and/or the progression of co-morbidities. Especially in the trajectory groups D and E the proportion of subjects with somatic and mental co-morbidities was high. In the two years after surgery, SA/DP levels continued to increase, most probably due to work disability related to the surgery, including recovery from the procedure and rehabilitation measures. The fact that work disability only started to decrease in the third year after surgery might also be due to complications related to the spine surgery. Severe side effects after spine surgery have been shown to occur in 10–24% of patients^8^. Possible complications include surgery-related infections, hematoma, nerve root injury and the risk of re-operation, or general complications like coronary ischemia, respiratory distress, and stroke^8^. The probability of re-operation within five years after decompression surgery has been shown to be 17% in a previous study^24^. However, we also saw an increase in work disability in about 7% in comparison group 1, and in about 4% in comparison group 2.

In both surgery cases and the two comparison groups, there was a trajectory group (E) with high levels of SA/DP (18% in cases and comparison group 1 and 8% in comparison group 2). A high proportion of individuals in this trajectory group suffered from mental co-morbidities measured as diagnosis-specific specialized health care and prescription of psychiatric medication. It has been shown, that mental disorders (particularly depressive and anxiety disorders), when co-occurring with chronic pain significantly increase the risk of work disability^3,12^, and of other negative clinical outcomes following lumbar spine surgery, ranging from low quality of life to higher mortality^23^.

We found that socio-demographic and clinical factors were differently associated with the respective trajectory groups. The best prognosis in terms of low levels of work disability had younger men with higher education who were married with children living at home, with no or low dosage of analgesic medication before surgery and no mental co-morbidity. These factors can be used to predict the prognosis for work disability in different patient groups with planned decompression surgery in the lumbar spine.

Applying group-based trajectory methodology, studies based on Swedish, Finnish or French cohorts have investigated patterns of work disability in individuals with diabetes mellitus, multiple sclerosis, and committing suicide^18,20,21^. The proportion of cases and subjects in comparison group 1 with constant high levels of work disability (trajectory groups E, 18%) was clearly lower compared to patients after a diagnosis of multiple sclerosis (28%)^18^, or subjects before suicide (30%)^21^, but clearly higher compared to subjects with diabetes mellitus^20^. The proportion of individuals in comparison group 2 with constant high levels of work disability (8%) in our study is similar to the proportion (9%) of people on disability pension in the general Swedish population^25^.

We found that younger age, male sex, higher education, living in big cities, being married cohabitating with children, no somatic or mental co-morbidity, and no analgesic, anxiolytic, hypnotic, or antidepressant medication the year before surgery were characteristics of trajectory group A and therefore associated with a good prognosis in terms of low work disability after lumbar spine surgery. These factors were also associated with the trajectory groups with low SA/DP in the two comparison groups (data not shown). This is in line with the literature, where younger age, better general and mental health, and lower baseline pain intensity were found to be associated with return to work in patients with low back pain, independently from having had spine surgery or not^26^.

To the best of our knowledge, this is the first study analyzing trajectories of work disability prior and after decompression surgery in patients with dorsopathies. The study comprised more than eight thousand patients – a large sample allowing statistical analyses with sufficient power. Another strength is the long period of observation, three years before and three years after surgery. The data used for the analyses (surgery codes, diagnoses, work disability, socio-demographic and clinical factors) from nationwide Swedish registers are of good quality without loss to follow-up^27^. The findings are generalizable to countries with similar health care and social insurance systems.

As a limitation it should be mentioned that information on SA for the first 14 days for employed individuals is not available. Thus, the length of work disability is likely to be underestimated. The comparison group 1 was identified based on inpatient care and specialized outpatient care in order to build an ideal comparison group to the cases, who also had such specialized health care. We did not include patients with dorsopathies treated in primary care only, or patients with back pain not seeking help at health care services. Thus, in interpreting the trajectory groups, it should be kept in mind that it is likely that only the most severe cases of back pain were included in this group. However, this can also be regarded as strength, since both cases and patients in comparison group 1 were derived from the same source population, guaranteeing good comparability. Accordingly, comparison group 2 might include patients with dorsopathies treated only in primary care or outside the health care system. Additionally, information on spine levels of decompression surgery was not available. The distribution of this variable across trajectory groups could therefore not be analysed in the logistic regression analyses. To get a more heterogeneous group, and to study incidental decompression surgery, individuals with a previous decompression surgery in the lumbar spine were excluded. However, it can be that study participants had had other spine surgery previously, which would make the study population more heterogeneous. Furthermore, despite the wide range of included covariates, other (not measured) socio-demographic and clinical factors such as health behavior and work-related factors^23,26,28^ can be associated with work disability in patients with dorsopathy and decompression surgery and should be investigated in future studies.

In this study we demonstrated that the group of patients with dorsopathy and decompression surgery was heterogeneous in terms of work disability prior to and after surgery. More than half of the patients had a good prognosis, i.e. very low levels or no work disability two years after surgery. The remaining patients had still 2, 4 and 8 months of work disability three years after surgery. Patterns of SA/DP before surgery, sex, socio-economic status, pain intensity and comorbid mental disorders provide crucial clinical information of work disability after the surgery.

These factors identified to predict work disability after lumbar spine surgery can be used for a tailored patient communication towards expectations of work ability after surgery. Furthermore, the knowledge gained from this study about future sickness absence and disability pension levels can be used by patients when planning their work as well as by health care professionals, and by social insurance officers. Employers and occupational health services can use this information to adequately handle workplace adjustments. Moreover, the information on the clinical and socio-demographic risk markers for long-term work disability can provide crucial information on the design of person-based rehabilitation measures.

## Conclusions

Only approximately half of the patients with dorsopathy had a favorable prognosis with low levels of work disability 3 years after decompression surgery. This suggests that a potential alleviation of symptom levels and pain through surgery is not necessarily associated with improvements in physical and occupational functioning, which in turn calls for strengthened vocational rehabilitation. Pre-surgery work disability, female sex, low (compulsory) education, a comorbid mental disorders and pain intensity provide crucial clinical information on work disability after surgery. These factors can guide the decision for intensified rehabilitation for particularly vulnerable groups. Reducing work disability has not only a strong effect on the affected patients’ lives, but also a clear economic component given the vast amounts of related social security expenses.

## Methods

### Study populations

Cases were defined as all individuals 25–60 years with a decompression surgery in the lumbar spine 2008–2010, who had a main diagnosis of a dorsopathy in specialized health care and were living in Sweden on 31^st^ of December the year before surgery (N = 8969). Excluded were individuals with decompression surgery in the lumbar spine three years before inclusion (N = 411). In total the study population comprised 8558 individuals. For comparative reasons two comparison groups were established. Comparison group 1: individuals with diagnosed dorsopathy (same diagnostic codes as the cases, based on main diagnoses in specialized health care), but without surgery 2008–2010 (n = 8558); comparison group 2: individuals from the general population without a diagnosed dorsopathy during 2001–2013 (N = 8558). Each individual from the comparison groups was matched to a case at the date of inclusion, i.e. decompression surgery (cohort entry date) by age, sex, educational level and region of birth. Individuals in both comparison groups had to live in Sweden the year before the cohort entry date.

### Data sources

The study was based on the IMAS (Insurance Medicine All Sweden) study^29^. The IMAS study comprises more than 9 million individuals 16 to 64 years of age resident in Sweden at end of 1984, 1989, 1994, 1999, 2004 or 2009 with retrospective and prospective data up to 2013, identified by registers from Statistics Sweden. Register data was available from the following three agencies: 1.) Statistics Sweden: sociodemographic factors, information on unemployment and emigration. 2.) The Social Insurance Agency: SA and DP (date and grade) from 2005 and onwards; 3.) The National Board of Health and Welfare: date and cause of in- and specialized outpatient care starting from 1973 and from 2001, respectively; date of death from 1961 and onwards and prescription of dispensed medication: date, defined daily doses (DDD) and Anatomic Therapeutic Chemical classification system (ATC) codes from July 2005 and onwards.

### Case definition

Cases with decompression surgery in the lumbar spine were defined according to the Swedish version of NOMESCO codes of surgical procedures: ABC 07, 16, 26, 36, 56, 66, 99.

### Diagnostics and covariates

All diagnoses derived from specialized health care visits were coded according to the International Classification of Diseases version 10 (ICD-10). Dorsopathies (main diagnoses measured at cohort entry date; main or side diagnoses measured three years before study entry) included following diagnoses: *Spondylopathy*: M47.8, M47.9, M47.9 K, M48.0, M48.0 K, M48.8, M48.8 K and M48.8 W; *Radiculopathy*: M51.1, M51.1 K, M54.1; and *Other dorsopathies*: M54.3 and M54.4, M53.8, M53.9, and M54.5. Other somatic disorders in specialized care (main diagnoses measured at cohort entry, main or side diagnoses measured three years before study entry) comprised ICD-10 chapters 1–4 and 6–19, except dorsopathic diagnoses (M40-M54) and uncomplicated birth (O80). Some cases had a fusion surgery in addition to the decompression surgery, which was coded as follows: NAG3, NAG4, NAG5, NAG6, NAG7, NAG8 and NAG 9.

Common mental disorders included depressive disorders (ICD-10 codes F32, F33), anxiety disorders (ICD-10 codes F40–42) and stress-related mental disorders (ICD-10 code F43). Other mental disorders comprised remaining ICD-10 codes of chapter 5, i.e. F00-F31, F34-F39 and F44-F99. Both groups of mental disorders were measured in specialized health care as main diagnoses at study entry and main or side diagnoses three years before study entry. Prescribed and dispensed medication for analgesics, anxiolytics, sedatives and antidepressants were coded according to the ATC codes N02A and N02B; N05B, N05C and N06A, respectively. Based on annual prescribed and dispensed daily doses (DDD) of medication in the year preceding inclusion, the prescriptions were divided into: 1) no dose (no DDD), 2) low dose (0–0.5 DDD, non-inclusive), 3) medium dose (0.5–1.5 DDD, inclusive) and 4) high dose (>1.5 DDD)^30^.

Socio-demographic factors included sex, age, educational level, family situation, type of living area, and region of birth country. All sociodemographic variables were measured at the end of the year before the cohort entry date. Moreover, work related factors were measured: unemployment (the calendar year preceding cohort entry date) and work disability 3 years preceding and 3 years following the cohort entry date. All covariates were categorized as shown in Tables 1 and 2. For cases with missing information on education and region of birth country, these observations were set to “Low (compulsory) education” and “Rest of the world”, respectively.

### Outcome measure

Work disability was measured as the annual sum of net months with SA or DP, and was measured 3 years before, and up to 3 years after surgery. We have chosen 3 years as the cut-off time in order to guarantee full coverage of information for outcome measures and covariates in the registers. “Net months” is considering the grade of SA and DP in a sense that if an individual has 50% SA/DP for 2 months, it will be counted as one net month.

### The Swedish social insurance system

The Swedish Social Insurance Agency pays sickness benefits to all people above the age of 16 with an income from work or unemployment benefits (also students), who have reduced work capacity due to disease or injury. During the first 14 days of SA, the employers are responsible for sick pay. There is one qualifying day without benefits, more for self-employed people. Physician certificate for the SA is required after 7 days of self-certification. DP can be granted to all people with permanent impaired work capacity due to disease or injury. For this analysis, work disability is defined, as being paid benefits due to work disability by the Swedish Insurance Agency. That means that for most employees, only sickness absence lasting longer than 2 weeks was considered.

### Statistical methods

Trajectories (patterns) were assessed with a Group Based Trajectory (GBT) method in terms of levels and trends of work disability, i.e. constant, increasing or decreasing trends at high, medium or low levels before and after decompression surgery^19^. The GBT modelling merely allows the identification of subgroups of individuals who follow distinct patterns of change over time and estimate the proportion of individuals in each group. The model is hereby able to capture the prognostic heterogeneity in patients with decompression surgery. The day of surgery (2008–2010) was defined as time point T0 and the patterns of SA/DP months (based on the annual mean) were measured annually from 2005–2013 (T-3 up to T + 3), i.e. during 6 time periods. The decision on the optimal number of trajectory groups was based on the Bayesian information criterion (BIC)^31^. Trajectories of annual work disability were also determined for both comparison groups defining T0 as the respective day of surgery of the matched case. In order to elucidate the work disability in more detail for the largest trajectory group in the cohort with decompression surgery, we also measured trajectories of SA/DP days per month in the year following surgery.

In addition, multinomial logistic regression was applied to assess the association of socio-demographic and clinical characteristics, including fusion surgery, (measured at and/or prior to T0) with the identified trajectory groups. The likelihood ratio chi^2^-test was used to detect differences in characteristics regarding the identified trajectories. Moreover, Nagelkerke R^2^ was assessed to evaluate the strength of these associations. We also measured the R^2^-value for each specific covariate (R^2^ difference) by removing each covariate from the full model. The higher the difference in R^2^, the more contributed the factor to the explanation of variance in the respective model. A p-value < 0.05 was considered significant.

### Ethical considerations

The study is based on several Swedish national registers, which are linked for research purposes. The analysis has been approved by the Regional Ethical Board of Karolinska Institutet. All registers used for this study were anonymised and de-identified prior to analysis by Statistics Sweden, which was responsible for data linkage. Thus, researchers received de-identified data. In Sweden, ethical vetting is always required when using register data and performed by regional review boards, and the risk appraisal associated with the Law on Public Disclosure and Secrecy is performed by the register keepers. Those ethical review boards can waive the requirement to consult the data subjects directly to obtain their informed consent. This is often the case if the research is supported by the ethical review board and the data have already been collected in some other context, e.g. routine data like insurance records.

## References

[1] Breivik H, Collett B, Ventafridda V, Cohen R, Gallacher D (2006). Survey of chronic pain in Europe: prevalence, impact on daily life, and treatment. Eur J Pain.

[2] Maher C, Underwood M, Buchbinder R (2017). Non-specific low back pain. Lancet.

[3] Dorner TE (2015). Sickness absence due to back pain or depressive episode and the risk of all-cause and diagnosis-specific disability pension: A Swedish cohort study of 4,823,069 individuals. Eur J Pain.

[4] Stromqvist B, Fritzell P, Hagg O, Jonsson B, Sanden B (2013). Swespine: the Swedish spine register: the 2012 report. Eur Spine J.

[5] Dagenais S, Tricco AC, Haldeman S (2010). Synthesis of recommendations for the assessment and management of low back pain from recent clinical practice guidelines. Spine J.

[6] Lurie J, Tomkins-Lane C (2016). Management of lumbar spinal stenosis. BMJ.

[7] Watters WC (2009). An evidence-based clinical guideline for the diagnosis and treatment of degenerative lumbar spondylolisthesis. Spine J.

[8] Zaina, F., Tomkins-Lane, C., Carragee, E. & Negrini, S. Surgical versus non-surgical treatment for lumbar spinal stenosis. *Cochrane Database Syst Rev*, CD010264, 10.1002/14651858.CD010264.pub2 (2016). 10.1002/14651858.CD010264.pub2 PMC6669253 26824399

[9] Weber H (1983). Lumbar disc herniation. A controlled, prospective study with ten years of observation. Spine (Phila Pa 1976).

[10] Weinstein JN (2009). Surgical compared with nonoperative treatment for lumbar degenerative spondylolisthesis. four-year results in the Spine Patient Outcomes Research Trial (SPORT) randomized and observational cohorts. The Journal of bone and joint surgery. American volume.

[11] Sumanen H, Pietilainen O, Lahti J, Lahelma E, Rahkonen O (2015). Interrelationships between education, occupational class and income as determinants of sickness absence among young employees in 2002-2007 and 2008-2013. BMC Public Health.

[12] Dorner TE (2016). Synergistic effect between back pain and common mental disorders and the risk of future disability pension: a nationwide study from Sweden. Psychol Med.

[13] Goesling J, Clauw DJ, Hassett AL (2013). Pain and depression: an integrative review of neurobiological and psychological factors. Curr Psychiatry Rep.

[14] Anderson JT (2015). Clinical depression is a strong predictor of poor lumbar fusion outcomes among workers’ compensation subjects. Spine (Phila Pa 1976).

[15] Jensen LD (2011). Predictors of vocational prognosis after herniated lumbar disc: a two-year follow-up study of 2039 patients diagnosed at hospital. Spine (Phila Pa 1976).

[16] Faour M (2016). Return to Work Rates After Single-level Cervical Fusion for Degenerative Disc Disease Compared With Fusion for Radiculopathy in a Workers’ Compensation Setting. Spine.

[17] Tye EY (2017). Decompression vs. Decompression and Fusion for Degenerative Lumbar Stenosis (DLS) in a Workers’ Compensation Setting. Spine.

[18] Björkenstam, C., Alexanderson, K., Wiber, M., Hillert, J. & Tinghög, P. Heterogeneity of sickness absence and disability pension trajectories among individuals with MS. *Multiple Slcerosis Journal*, 1–11, 10.1177/205521731559563 (2015). 10.1177/2055217315595638 PMC5433496 28607698

[19] Jones B, Nagin D, Roeder K (2001). SAS procedure on mixture for estimating development trajectories. Sociological Methods & Research.

[20] Virtanen M (2015). Lifestyle-related risk factors and trajectories of work disability over 5 years in employees with diabetes: findings from two prospective cohort studies. Diabet Med.

[21] Wang M (2015). Trajectories of Work-Related Functional Impairment prior to Suicide. PLoS One.

[22] Cobo Soriano J (2010). Predictors of outcome after decompressive lumbar surgery and instrumented posterolateral fusion. Eur Spine J..

[23] Wilson CA, Roffey DM, Chow D, Alkherayf F, Wai EK (2016). A systematic review of preoperative predictors for postoperative clinical outcomes following lumbar discectomy. The spine journal: official journal of the North American Spine Society.

[24] Lad SP, Babu R, Ugiliweneza B, Patil CG, Boakye M (2014). Surgery for spinal stenosis: long-term reoperation rates, health care cost, and impact of instrumentation. Spine.

[25] Social insurance agency. *Social insurance in figures*. (2017).

[26] Grovle L (2013). Prognostic factors for return to work in patients with sciatica. The spine journal: official journal of the North American Spine Society.

[27] Ludvigsson JF (2011). External review and validation of the Swedish national inpatient register. BMC Public Health.

[28] Mittendorfer-Rutz E, Dorner TE (2018). Socio-economic factors associated with the 1-year prevalence of severe pain and pain-related sickness absence in the Austrian population. Wien Klin Wochenschr.

[29] Mittendorfer-Rutz E (2012). Sickness absence due to specific mental diagnoses and all-cause and cause-specific mortality: a cohort study of 4.9 million inhabitants of Sweden. PLoS One.

[30] Tiihonen J, Mittendorfer-Rutz E, Torniainen M, Alexanderson K, Tanskanen A (2016). Mortality and Cumulative Exposure to Antipsychotics, Antidepressants, and Benzodiazepines in Patients With Schizophrenia: An Observational Follow-Up Study. Am J Psychiatry.

[31] White DK, Neogi T, Zhang Y, Niu J, Katz PP (2017). Association of Slow Gait Speed With Trajectories of Worsening Depressive Symptoms in Knee Osteoarthritis: An Observational Study. Arthritis Care Res (Hoboken).

